# The market values of Chinese energy firms during COVID-19 pandemic

**DOI:** 10.1007/s44176-023-00011-w

**Published:** 2023-02-21

**Authors:** Lu Shi

**Affiliations:** 1 School of Management, Wuhan University of Technology, Wuhan, 430070 Hubei China; 2 Research Institute of Digital Governance and Management Decision Innovation, Wuhan University of Technology, Wuhan, China

**Keywords:** Cumulative abnormal return, COVID-19, Energy firm, Corporate social responsibility, Lockdown policies, G12, G14, G32, I10

## Abstract

This paper studies the impact of coronavirus disease 2019 (COVID-19) on Chinese energy firms’ market values by using event study approach. First, we find that the cumulative abnormal return (CAR) of energy firms significantly decreases 2.7–10.6% on average due to the negative shock of COVID-19 on energy market. Second, we present that Corporate Social Responsibility (CSR) performance could significantly reduce the negative market reaction of energy firms during COVID-19. Finally, in using the intervention policy of various cities as exogenous shocks, we provide evidence that stock returns of energy firms significantly increase after cities where firms located in issued lockdown policies.

## Introduction

The suddenly shocks of COVID-19 pandemic swept the globe in 2020, which led to a new round of the global economic crisis (Maliszewska et al. 2020; Padhan and Prabheesh 2021). As the International Monetary Fund (IMF) forecasted, global economy in 2020 was expected to contract sharply by − 3% because of COVID-19 pandemic, which was much worse than the effect of 2008 financial crisis. The world across all countries and industries suffered enormous and heterogeneous economics losses. For example, due to the outbreak of COVID-19, stock prices of the US firm in manufacturing industry decreased by 29% (Ding et al. 2021). S&P 500 implemented circuit breakers thrice in March 2020. 43% of small businesses in the U.S. are temporarily closed (Bartik et al. 2020). 214 cities in China experienced significant consumption decreases ranging from 14 to 69% (Chen et al. 2021).

COVID-19 disease is highly contagious in the public, resulting in mass cases infected within a short time. At the initial outbreak of pandemic, modern medicine had little impact in this new special of virus, COVID-19 disease. Before the vaccine aimed at COVID-19 disease was developed, most countries had no choice but to require the public keeping social distance to prevent spreading this virus, which even decided to shut down the plants and seal off the city (Fang et al. 2020). Accordingly, the outbreak of COVID-19 and the implementation of lockdown policies restricted human mobility, led to economics activities hindered. It greatly reduced energy consumptions across the world, thereby have a big influence on the demand of energy, especially for China that is the largest energy importer and consumer around the world.

Assessing the economics consequence of COVID-19 pandemic attracted more attention from government and scholars. A rapidly growing of studies focused on the economics consequence of COVID-19 on stock market. For example, compared with previous serious infectious disease and major events in the US stock market, Baker et al. (2020) documented this unprecedented market reaction during COVID-19. Using the infected cases to forecast the next-day stock market reaction, Alfaro et al. (2020) studied the relationship between market value losses during COVID-19 and firm characteristics. Then based on the role of firm characteristics, most research aimed to study the resiliency of different firms when faced this unprecedented pandemic (Bartik et al. 2020; Ding et al. 2021). There is part of researches that tended to investigate the heterogeneous effect of COVID-19 among different industries, such as insurance market (Wang et al., 2020), consumption industry (Chen et al. 2021), solar industry (Wei et al., 2021). He et al. (2020) compared stock price among different sectors during COVID-19, however not to design detailed and precise research to in-depth study the heterogeneous shocks of COVID-19 among different industries.

We focus on the strand of the literature about the economics consequence of COVID-19 on energy firm. Financial market and energy market is two important strands in the related research about the consequence of COVID-19 (Padhan and Prabheesh 2021).^1^ As kind of essential production factor, energy market has always been a cynosure during COVID-19 and a hot topic in academic research, including oil price (Jia et al. 2021; Ren et al. 2021; Zhang and Hamori 2021), energy firms (Huang and Liu 2021; Si et al. 2021). Due to the sudden shocks of COVID-19 on energy demand and supply, we could expect that the reduction of economic activities following COVID-19 may have obvious influence on energy firm values, thus eventually affecting market value. Thus, we use the outbreak of COVID-19 to investigate the market reaction of Chinese energy firm caused by COVID-19.

With the spread of COVID-19 pandemic, the expectation that human mobility restrictions and blocked economics activities results in the faltering demand of energy, coupled with the expected economic recession during COVID-19, greatly affects energy’ supply and demand and severely hit energy industry. Meanwhile, as the top importer and consumers of energy around the world, China’s economy have an obvious characteristic of high energy dependence and energy consumption. The strong price fluctuations of energy in international market have unintended impact on the price adjustment of energy from the China government, which energy price is regulated in China. Based on the above discussion, the slump in international demand of energy caused by COVID-19 and domestic uncertain oil price policy in China would exert negative effect on energy industry. Thus, we postulate that energy firms suffered the more severe market value losses than other firms under multiple-pronged attacks caused by COVID-19.

The rationale that we focus on Chinese energy firms is as follows: Firstly, there is growing consensus that the supply and demand of energy is greatly focused by government, firms, and consumers. Particularly, China is the world’s biggest importer and consumer of energy. The change of economics situation home and abroad is closely related to Chinese energy market. Secondly, China was the first to suffer the outbreak of COVID-19 and issue lockdown policies around the world. This shock on Chinese stock market that we identify could not be disturbed by spillover effect of COVID-19 from other countries, thereby providing the most suitable setting to study the shock of COVID-19. Thirdly and most importantly, it is widely acknowledged that energy is crucial material basis of the development of economics. Given that this unprecedented pandemic was abrupt and far-reaching, all industries almost were involved. As the important fundamental industry, the recession of other industries would indirectly affect the development of energy firms. Hence, the losses of energy firm could better capture the shock of this pandemic on China’s economy.

To conduct an empirical analysis, we measure short-term market reaction of energy firms during COVID-19 by using event study approach, to capture the timely change of energy firms’ market values during COVID-19. Specifically, we first define the date of the Wuhan lockdown^2^ as the outbreak of COVID-19. On this day, COVID-19 pandemic increasingly attracted concerns from China, even the world. Hence, we regard January 23 of 2020 (the date of Wuhan lockdown) as event date, and then calculate the cumulative abnormal returns (*CAR*). Second, to identify the net market reaction caused by COVID-19, we use the *CAR* in a five day before and after COVID-19 to measure market reaction, thereby investigating the change of energy firms’ market values by exploring the difference between before and after COVID-19.

To reduce the bias caused by firm characteristics, we adopt difference and differences (DID) approach to compare the difference between treatment and control group, thereby identifying the net losses of energy firms during COVID-19. First, based on the different control group, we show that the shareholders’ wealth of energy firms significantly reduces by 2.7–10.6% due to the outbreak of COVID-19. After the robustness check, we get consistent results that COVID-19 pandemic significantly decreases the stock return of energy firms, including using daily abnormal return (*AR*) in a five day before and after COVID-19 and changing the event windows to re-measure CARs.

Second, based on the importance of corporate social responsibility (CSR) activities when firms suffered negative events (Kong 2012; Lins et al. 2017; Kong et al. 2019; Albuquerque et al. 2020), we study how CSR performance affects energy firms’ market values during COVID-19. Our results show that CSR performance could moderate this negative market reactions of energy firms and soften market value losses during COVID-19. Finally, to evaluate the effectiveness of intervention policies, we use this exogenous shock as basis to study the market reaction caused by lockdown policies during the COVID-19 pandemic. According to the lockdown date of city in where the listed firm located, we set the lockdown day as event day, and calculating CAR, which we exclude the listed firms in cities where hadn’t issued the lockdown policies. We present evidence that lockdown policies during COVID-19 significantly reduce the value loss of energy firms.

We contribute to the literature on the loss of firm value related to COVID-19 by demonstrating the negative market reactions during COVID-19. Compared with prior studies that only investigated the unprecedented stock market during COVID-19 in all industries (Alfaro et al. 2020; Baker et al. 2020), this paper focuses more on the heterogeneous effect of energy industry during COVID-19. Coupled with the importance of energy on China’ economy, we particularly concentrate on the market reaction of energy firms during COVID-19 and mainly complement the literature on the heterogeneous effect of COVID-19 across different industries.

This paper also enriches the literature about the role of CSR on firms during crisis (Kong 2012; Lins et al. 2017; Kong et al. 2019; Albuquerque et al. 2020). Our findings that better CSR performance suffer the less loss during COVID-19 supports the importance of CSR when suffering adverse shocks. Then we study how the lockdown policies positively affect the stock market, which may reduce the panic about COVID-19, which provides clear policy implications for government to respond to the pandemic. Namely the intervention policy aimed at pandemic not always harms economy.

The remainder of the paper is as follows. Section 2 presents the data and research design, consisting of samples selections, variables definitions, and research design. Section 3 shows empirically results, including and descriptive statistics, baseline results, robustness checks, the effect of listed firms’ CSR performance, and the test on lockdown policies. Finally, we conclude this paper in Sect. 4.

## Data and research design

### Samples

Based on the selection of energy firms, we include all listed energy firms that is collected in State Intellectual Property Office (SIPO) database, which are 248 energy firms. Our sample also consists of 3,445 non-energy firms that is listed in Chinese stock market. Then this paper mainly conducts the following steps to handing data. First, we delete the firms that total assets are less than total debts. Then we exclude firms marked “ST (Special Treatment)”, which is at the loss and may be forced to delist, and the firms in financial industry. Third, we exclude listed firms with missing data and winsorize continuous variables at the 1st and 99th. Finally, we obtain empirically sample that includes 198 energy firms and 2352 non-energy firms.

Our data, including information on the stock returns of firms, and financial statements, mainly is from China Stock Market & Accounting Research (CSMAR) Database. Noticeably, the daily stock returns in this paper have included the cash dividend reinvestments. Our CSR data is from Rankins rating (RKS) that is the most authoritative and specialized database in China.

### Main variables

#### Cumulative abnormal return

Event study method is always applied to measure the shocks of exogenous and special events on listed firms’ market return in stock market (Kong 2012; He et al. 2020; Ding et al. 2021), which has been widely used in the research of financial and accounting fields. Thus, this paper captures the market reactions during COVID-19 by using event study method.

First, we set the day of outbreak of COVID-19 as the event day, which is marked as ${T}_{0}$ (${T}_{0}=0$). Then, we conduct the capital assert pricing model (CAPM) to obtain the estimated coefficient ${\widehat{\alpha }}_{i}$ and ${\widehat{\beta }}_{i}$, which the estimation window is [T_0_ − 180, T_0_ − 30].

1 $${Return}_{i,j}={\widehat{\alpha }}_{i}+{\widehat{\beta }}_{i}{Market Return}_{j}+{\epsilon }_{i,j}$$

where ${Return}_{i,j}$ is real daily stock return of each firm $i$ on day $j$. ${Market Return}_{t}$ refers to market return that is the sum of real daily stock return of all listed firms on day $j$, which traded market value of firms is regarded as the weight.

Second, we use Model (2) to obtain abnormal return (${AR}_{i,j}$) by real daily stock return minus expected daily stock return estimated by Model (1)*.* Noticeably, the expected daily stock return refers to the daily stock return if there is no shock of event, suggesting that the value of ${AR}_{i,j}$ equal zero without special events occurred.

2 $${AR}_{i,j}={Return}_{i,j}-\left(\widehat{\alpha }+\widehat{\beta }{Market Return}_{j}\right)$$

Finally, we total the abnormal return (${AR}_{i,j}$) during the period of event window [T_1_, T_2_] to obtain the cumulative abnormal return during the period of [T_1_, T_2_] (${CAR}_{i}\left[{T}_{1},{T}_{2}\right]$) for each firm $i$ as the Model (3). Here, *CAR* refers to the overall financial market responses to the shock.

3 $${CAR}_{i}\left[{T}_{1},{T}_{2}\right]={\sum }_{{T}_{1}}^{{T}_{2}}{AR}_{i,j}$$

In this paper, we use the *CAR* over the event windows [T_0 _− 5, T_0 _− 1] and [T_0_ + 1, T_0_ + 5] to measure the market reaction before and after COVID-19, respectively.

#### Corporate social responsibility performance

Our CSR variable comes from Rankins rating (RKS). As being increasingly aware of the importance of CSR, the listed firm in China pay more attention on CSR activities. The RKS reports that almost 851 listed firms in 2018 conducted CSR activities and issued related details in the independent CSR report, which increased 129% from a year earlier and continues to grow since 2009.^3^ Then based on the CSR activities of listed firms in each year, the RKS would evaluate their CSR performance and assign the scores from 0 to 100 for CSR performance of listed firms. Compared with the value of CARs, the value of CSR is too large, then causing bias on our regression result. Thus, this paper uses the natural logarithm of CSR scores to capture the degree of firms’ CSR performance. Additionally, we assign the CSR performance as 0 for the firm without conducting CSR activities and independent annual reports to issue the CSR activities.

#### Control variables

Based on previous market reaction studies (Kong 2012; He et al. 2020; Ding et al. 2021), the following control variables that maybe affect the stock returns are included in our regression model. For example, *Size* is the natural logarithm of total market value, which is used to control the impact of firm size. *Leverage* represents financial leverage that is total debts divided by book value of total assets. *Roe* is calculated as the net profits scaled by net assets. *MB* is firms’ growth that is calculated as total market value scaled by book value of total assets (Table 1).

**Table 1:** The definition of variables

Variables	Definition
*CAR*	The cumulative abnormal return of the listed firm in a 5 days before and after the outbreak of COVID-19, respectively, to measure the market reactions
*CAR7*	The cumulative abnormal return of the listed firm in a 7 days before and after the outbreak of COVID-19, respectively, to measure the market reactions
*AR*	The daily abnormal return during the period of [T_0 _− 5, T_0_ + 5]
*Treat*	1 for the 2020 data of energy firms, and 0 for others
*Post*	1 for the period after COVID-19 (namely, January 23 of 2020), and after the corresponding period of previous year in lunar calendar, and 0 otherwise
*Size*	Firm size, which is the natural logarithm of firm’ market value
*Leverage*	Financial leverage, which is the ratio of total liabilities to total assets
*Roe*	Return of equity, which is the ratio of net profits to net assets
*MB*	Firm growth, which is total market value divided by total assets
*CSR*	Corporate Social Responsibility Performance, which is the natural logarithm of rating scores of firms’ CSR performance

### Research design

Referring the studies of Liu et al. (2020), we employ DID method to study the shocks of COVID-19 on stock market, which conducts two research design by using different control groups.

First, we mainly compare the energy firm and non-energy firms during the same period to study the impact of COVID-19. Under this setting, ${Treat}_{i}$ takes value 1 for the 2020 data of energy firms, and 0 for the 2020 data of non-energy firms. ${Post}_{t}$ equals 1 for after January 23 in 2020, and 0 otherwise. Thus, the variable ${Treat}_{i}*{Post}_{t}$ captures the impacts of COVID-19 on energy firms’ market values relative to non-energy firms as the same period.

Second, we define the energy firms in 2020 that suffered the outbreak of COVID-19 as treatment groups, and set the same energy firms in 2019 without being involved by COVID-19 as control groups. Specifically, based on the different period of energy firm, ${Treat}_{i}$ equals 1 for the energy firm in 2020, and 0 for the same energy firm in 2019. Accordingly, ${Post}_{t}$ equals 1 after January 23 in 2020, and after the corresponding period of previous year in lunar calendar,^4^ and 0 otherwise. Hence, the variable ${Treat}_{i}*{Post}_{t}$ measures the change of energy firms’ market values during COVID-19 by comparing with themselves at the previous year. The empirically specification is as Model (4).

4 $${CAR}_{i,t}={\alpha }_{i}+{\beta }_{1}{Treat}_{i}*{Post}_{t}+{\beta }_{2}{Post}_{t}+{\beta }_{3}{Treat}_{i}+{\beta }_{4}{Size}_{t-1}+{\beta }_{5}{Leverage}_{t-1}+{\beta }_{6}{Roe}_{t-1}+{\beta }_{7}{MB}_{t-1}+Industry+{\epsilon }_{i,t}$$

where the variable ${CAR}_{i,t}$ is cumulative abnormal return in the five days before or after COVID-19 according to the variable ${Post}_{t}$. For stock return regression, we control for *Size*, *Leverage*, *Roe*, *MB*, and industry fixed effects.

## Estimation result

### Descriptive statistics and univariate analysis

Table 2 reports the summary statistics. First, the mean of the variable *CAR* is − 0.004 in Panel A, revealing that more than half of listed firms get a negative stock return. The average of the variable *CAR7* (mean = − 0.003) and *AR* (mean = − 0.001) also support this result. Second, Panel B reports the stock return of energy firm before the outbreak of COVID-19. The three kind of stock return suggest that 25% of energy firms at least have a positive stock return before COVID-19. Third, according to the 75th percentile of *CAR* in Panel C, we find that nearly three quarter of energy firms have a negative stock return after COVID-19. Based on the difference between stock return before and after COVID-19, both results primary show that stock return after COVID-19 is less than compared with stock return before COVID-19.

**Table 2:** Summary statistics

	Obs	Mean	Std. dev.	Min	P25	Median	P75	Max
Panel A. All sample								
* CAR5*	5488	− 0.004	0.072	− 0.142	− 0.047	− 0.014	0.019	0.310
* CAR7*	5488	− 0.003	0.067	− 0.141	− 0.045	− 0.013	0.022	0.247
* AR*	30,184	− 0.001	0.030	− 0.085	− 0.014	− 0.002	0.010	0.097
* Size*	2744	22.64	0.994	21.16	21.91	22.43	23.21	25.84
* Leverage*	2744	0.441	0.194	0.0670	0.292	0.434	0.587	0.866
* Roe*	2744	0.059	0.128	− 0.700	0.030	0.068	0.114	0.303
* MB*	2744	1.660	0.956	0.795	1.078	1.355	1.857	6.392
Panel B. Pre-pandemic of energy firms in 2020								
* CAR[− 5,− 1]*	188	− 0.009	0.042	− 0.142	− 0.024	− 0.009	0.004	0.310
* CAR[− 7,− 1]*	188	− 0.008	0.044	− 0.141	− 0.028	− 0.011	0.004	0.247
* AR*	940	− 0.002	0.016	− 0.085	− 0.008	− 0.003	0.004	0.091
*Panel C. Post-pandemic of energy firms in 2020*								
* CAR[1,5]*	188	− 0.042	0.057	− 0142	− 0.072	− 0.045	− 0.020	0.310
* CAR[1,7]*	188	− 0.035	0.050	− 0.141	− 0.066	− 0.038	− 0.013	0.215
* AR*	1128	− 0.006	0.027	− 0.085	− 0.017	− 0.005	0.007	0.097

Panel A presents the descriptive statistics. Panel B presents market reaction of energy firms in 2020 before the outbreak of COVID-19. Panel C present the market reaction of energy firms in 2020 after the outbreak of COVID-19. The definition of all variables is presented in Table 1

**Fig. 1:**
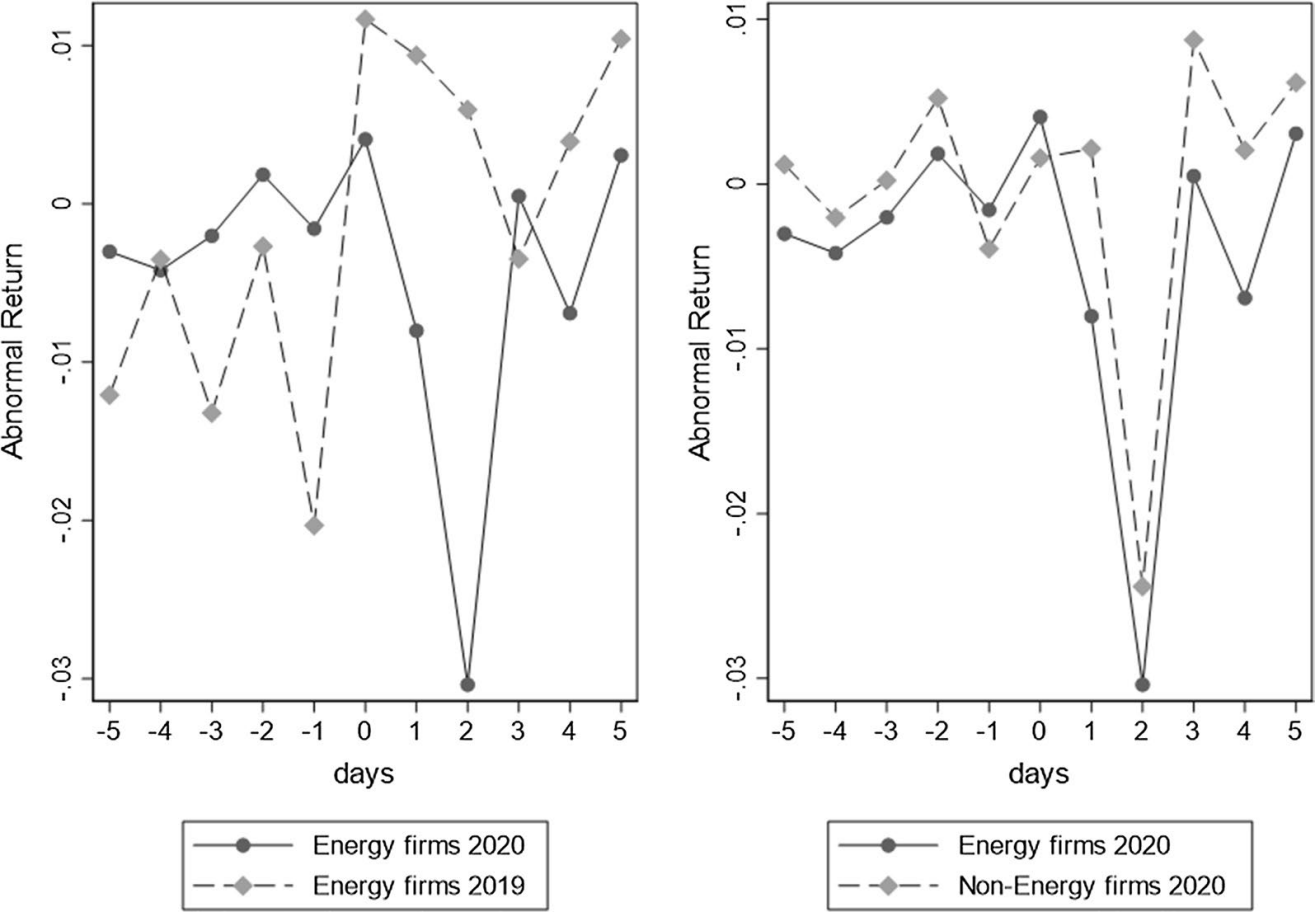
The market reaction of energy firms and different control group in event windows. This figure describes the daily abnormal return of energy firms and different control group during the event windows of [T_0 _− 5, T_0_ + 5], which ${{\varvec{T}}}_{0}$ is regarded as the date of the outbreak of COVID-19. X-axis is defined as the timeline according to the outbreak of COVID-19. Y-axis represents the average of daily abnormal returns

Figure 1 describes the daily stock return of energy firms and different control groups during the event windows of [T_0 _− 5, T_0_ + 5], in which we use the mean of daily abnormal return to observe the average effect of COVID-19 in treatment and different control group. As shown in right graph of Fig. 1, the daily average *ARs* of energy firms and non-energy firms in 2020 begin to decrease after the outbreak of COVID-19, and last for two days. Right graph illustrates three key point. First the *ARs* is insignificantly different with zero during the period of [T_0 _− 5, T_0_], suggesting that our CAPM model used to calculate the *ARs* is effective.^5^ Second the period of stock price crash ([T_0_ + 1, T_0_ + 2]) is matched with the actual day of outbreak of COVID-19. Finally, the shocks of COVID-19 on Chinese financial market almost involve in all listed firms.

The left graph in Fig. 1 includes the *ARs* of the energy firms during the outbreak of COVID-19 and during the corresponding period of previous year in lunar calendar. The result show that the energy firms in 2019 keep small positive ARs in the day of [T_0_, T_0_ + 2], and that the energy firms in 2020 that experience the shock of COVID-19 have greater loss than energy firms in 2019 without outbreak of COVID-19 pandemic. Obviously, energy firms receive a greater loss than non-energy firms after COVID-19.

### Baseline results

Table 3 shows the regression result from estimating Model (4) under two different control groups. First, we find the coefficients of Treat*Post are significantly negative in column (1) and (2), suggesting that stock return of energy firms decreases by 10.6% relative to the previous year because of the shock of COVID-19. Second, when comparing energy firms to non-energy firms in column (3) and (4), we get constant results that the COVID-19 incurs an additional 2.7% decreased in CAR or the shareholders’ wealth compared with other listed firms in 2020. Third, in control variables, we find firm size and earnings performance are positively associated with *CAR*, implying that firms with large size and better profitability have stronger resilience to COVID-19 shock and suffer the less loss in stock return.

**Table 3:** Baseline results

Treatment group Control group	*CAR*			
	Energy firms 2020		Energy firms 2020	
	Energy firms 2019		Non-energy firms 2020	
	(1)	(2)	(3)	(4)
Treat*Post	− 0.106***	− 0.106***	− 0.027***	− 0.027***
	(0.008)	(0.007)	(0.006)	(0.005)
*Post*	0.073***	0.073***	− 0.006***	− 0.006***
	(0.006)	(0.005)	(0.002)	(0.002)
*Treat*	0.038***	0.037***	− 0.002	− 0.002
	(0.005)	(0.005)	(0.004)	(0.004)
*Size*		0.004**		0.006***
		(0.002)		(0.001)
*Leverage*		0.009		0.002
		(0.013)		(0.006)
*Roe*		0.038*		0.025***
		(0.021)		(0.009)
*MB*		− 0.006		0.000
		(0.005)		(0.001)
*Industry effects*	Yes	Yes	Yes	Yes
*Constant*	− 0.050***	− 0.153***	− 0.021***	− 0.155***
	(0.005)	(0.050)	(0.007)	(0.023)
*Obs*	764	764	5100	5100
*R2*	0.239	0.251	0.067	0.076

The table reports the impact of COVID-19 on energy firms’ market values. Dependent variable is cumulative abnormal returns (*CAR*) in a 5 days before and after COVID-19 to capture the change of market values. Adjusted robust standard errors are in parentheses. The definition of other variables is shown in Table 1. ***, **, and * is regarded as significance at the level of 1%, 5%, and 10%, respectively

Based on the above results, we demonstrate that the outbreak of COVID-19 could significantly reduce market value of Chinese energy firms by 2.7–10.6% on average.

### Robustness tests

First, we use the daily *AR* in the event windows [T_0 _− 5, T_0_ + 5] to measure the market reaction during COVID-19, which the result is shown as Panel A of Table 4. When using the daily *ARs*, we control fixed effect of the date on the basis of Model (4) to conducts the regression. The coefficients of Treat*Post are statistically significant and negative, which are consistent under different control variables and different control groups. It reveals that the daily AR on energy firms decreases by 0.4–2.1% after the outbreaks of COVID-19.

**Table 4:** Alternative market reaction: *AR* and* CAR7*

Treatment group Control group	Energy firms 2020		Energy firms 2020	
	Energy firms 2019		Non-energy firms 2020	
Panel A. The daily abnormal return				
Variables	*AR*			
	(1)	(2)	(3)	(4)
Treat*Post	− 0.021***	− 0.021***	− 0.004***	− 0.004***
	(0.001)	(0.001)	(0.001)	(0.001)
*Post*	0.017***	0.025***	− 0.001**	0.005***
	(0.001)	(0.002)	(0.000)	(0.001)
*Treat*	0.009***	0.008***	− 0.000	− 0.000
	(0.001)	(0.001)	(0.001)	(0.001)
*Size*		0.000		0.001***
		(0.000)		(0.000)
*Leverage*		0.002		0.001
		(0.002)		(0.001)
*Roe*		0.009**		0.005***
		(0.004)		(0.002)
*MB*		− 0.002*		0.000
		(0.001)		(0.000)
*Industry and date effects*	Yes	Yes	Yes	Yes
*Constant*	− 0.011***	− 0.023**	− 0.005***	− 0.024***
	(0.001)	(0.009)	(0.002)	(0.004)
*Obs*	4,202	4,202	28,050	28,050
*R2*	0.069	0.122	0.013	0.014

*Panel B. The different period of cumulative abnormal return*				
*Variables*	*CAR7*			
	(1)	(2)	(3)	(4)
Treat*Post	− 0.102***	− 0.102***	− 0.014***	− 0.014***
	(0.008)	(0.008)	(0.005)	(0.005)
*Post*	0.075***	0.075***	− 0.013***	− 0.013***
	(0.006)	(0.006)	(0.002)	(0.002)
*Treat*	0.038***	0.038***	− 0.008*	− 0.008**
	(0.005)	(0.005)	(0.004)	(0.004)
*Size*		0.003		0.005***
		(0.002)		(0.001)
*Leverage*		0.011		0.005
		(0.014)		(0.006)
*Roe*		0.012		0.039***
		(0.029)		(0.009)
*MB*		− 0.005		− 0.000
		(0.006)		(0.001)
*Industry effects*	Yes	Yes	Yes	Yes
*Constant*	− 0.052***	− 0.114**	0.001	− 0.118***
	(0.005)	(0.054)	(0.009)	(0.022)
*Obs*	764	764	5,100	5,100
*R2*	0.215	0.216	0.058	0.071

The table shows the robustness results by using alternative the measurement of market reaction. Panel A report the results of the daily abnormal return (*AR*) in the event windows *[T*_*0*_*− 5, T*_*0*_*− 5]*. The result using cumulative abnormal return (*CAR7*) of the listed firm in a 7 days before and after COVID-19 is shown in Panel B, which *CAR* in pre-COVID is calculated in event windows *[T*_*0*_*− 7, T*_*0*_*− 1]*, and the *CAR* in post-COVID is measured in event windows *[T*_*0*_ + *1, T*_*0*_ + *7]*. Adjusted robust standard errors are in parentheses. The definition of other variables is reported in Table 1. ***, **, and * is regarded as significance at the level of 1%, 5%, and 10%, respectively

Next, we use *CAR[− 7,− 1]* or *CAR[1,7*] to measure the *CAR* before and after COVID-19, then re-conduct empirically regression, which is shown in Panel B of Table 4. Based on the coefficients of Treat*Post, we find that *CAR* of energy firms decreases by 1.4%-10.2% after the outbreaks of COVID-19. Overall, with the different measures of market reaction, we get consistent results that COVID-19 significantly decrease the stock return of energy firms.

### CSR performance

In this section, we investigate how CSR performance affects the loss of energy firms’ market value during COVID-19. Existing literature has studied the role of CSR on firms’ value (Kong 2012; Deng et al. 2013; Lins et al. 2017; Albuquerque et al. 2019; Kong et al. 2019). Given that CSR activities contribute the firm to build trust with stakeholders (Deng et al. 2013), the firm with better CSR performance have the less drop in stock return when firms suffer adverse event, such as the melamine contamination incident (Kong 2012), the 2008 financial crisis (Lins et al. 2017), food recall events because of food safety incidents (Kong et al. 2019). Hence, we expect that the better CSR performance prior pandemic can decrease the value loss of energy firms during COVID-19.

Specifically, we use the variable *CSR* that the data is from RKS, which the higher value of variable *CSR*, the better of CSR performance. Also, the value of *CSR* equals 0 for the firm without conducting CSR activities, the value of CSR equals 0. Then we estimate it by following model:

5 $${CAR}_{i,t}={\alpha }_{i}+{\beta }_{1}{Treat}_{i}*{Post}_{t}*{CSR}_{i,t-1}+{\beta }_{2}{Treat}_{i}*{Post}_{t}+{\beta }_{3}{Treat}_{i}*{CSR}_{i,t-1}+{\beta }_{4}{Post}_{t}*{CSR}_{i,t-1}+{\beta }_{5}{Treat}_{i}+{\beta }_{6}{Post}_{t}+{\beta }_{7}{CSR}_{i,t-1}+{\beta }_{8}{Size}_{t-1}+{\beta }_{9}{Leverage}_{t-1}+{\beta }_{10}{Roe}_{t-1}+{\beta }_{11}{MB}_{t-1}+Industry+{\epsilon }_{i,t}$$

where the variable ${Treat}_{i,t}*{Post}_{i,t}*{CSR}_{i,t-1}$ captures the impact of CSR of the level of each listed firms and how CSR affect the market reaction during COVID-19. Relative to the value of *CAR*, the value of CSR is too large, which easily results in regression bias and that the coefficients of ${Treat}_{i,t}*{Post}_{i,t}*{CSR}_{i,t-1}$ is too small. Thus, we use the logarithm of *CSR* to run regression. Table 5 reports the results by using Model (5).

**Table 5:** Effects of corporate social responsibility

Treatment group Control group	*CAR*			
	Energy firms 2020		Energy firms 2020	
	Energy firms 2019		Non-energy firms 2020	
	(1)	(2)	(3)	(4)
Treat*Post*CSR	0.023***	0.023***	0.005*	0.005*
	(0.004)	(0.004)	(0.003)	(0.003)
Treat*Post	− 0.135***	− 0.135***	− 0.034***	− 0.034***
	(0.009)	(0.009)	(0.007)	(0.007)
Treat*CSR	− 0.010***	− 0.010***	0.000	0.000
	(0.003)	(0.002)	(0.002)	(0.002)
Post*CSR	− 0.018***	− 0.018***	− 0.001	− 0.001
	(0.003)	(0.003)	(0.001)	(0.001)
*Treat*	0.050***	0.049***	− 0.002	− 0.002
	(0.007)	(0.006)	(0.005)	(0.005)
*Post*	0.096***	0.096***	− 0.005**	− 0.005**
	(0.007)	(0.007)	(0.003)	(0.003)
*CSR*	0.010***	0.009***	0.001	− 0.001
	(0.002)	(0.002)	(0.001)	(0.001)
*Size*		0.004		0.006***
		(0.002)		(0.001)
*Leverage*		0.009		0.002
		(0.013)		(0.006)
*Roe*		0.039*		0.024***
		(0.020)		(0.009)
*MB*		− 0.005		0.000
		(0.005)		(0.001)
*Industry effects*	Yes	Yes	Yes	Yes
*Constant*	− 0.064***	− 0.150***	− 0.021***	− 0.166***
	(0.006)	(0.052)	(0.007)	(0.025)
*Obs*	764	764	5,100	5,100
*R2*	0.280	0.289	0.067	0.077

The table presents the effect of corporate social responsibility on energy firms’ market values during COVID19. Dependent variable is cumulative abnormal returns (*CAR*) in a 5 days before and after COVID-19 to capture the change of market values. *CSR* is natural logarithm of corporate social responsibility performance of listed firms. Adjusted robust standard errors are in parentheses. The definition of other variables is reported in Table 1. ***, **, and * is regarded as significance at the level of 1%, 5%, and 10%, respectively

When comparing with the same energy firms in previous year, the coefficients of Treat*Post*CSR are positive and statistically significant, revealing that better CSR performance prior can mitigates the value loss due to COVID-19. Then comparing energy firms to non-energy firms in 2020 in column (3) and column (4), we get constant results.^6^ Consequently, both estimates imply that energy firms that perform better in CSR activities or investing more for CSR activities prior to the pandemic, suffer the less drop in market value during COVID-19.

### Lockdown policies

To respond to COVID-19, the Chinese government decided to issue the lockdown policies for parts cities, including maintain social distancing, undergo self-isolation, and even sealed off the city. This lockdown policy issued is unexpected and rapid for everyone, which can be regarded as a quasi-natural experiment. Thus, we use the exogenous shock of lockdown policy as basis to study the market reaction of energy firms during COVID-19. According to the lockdown date of city in where the listed firm located,^7^ we set the day as event day, and calculating *CAR[− 5,− 1]* and *CAR[1,5]* to reflect the market reactions before and after COVID-19. For the 2019 data, we use the corresponding lunar calendar date in 2019 to measure the stock return. We exclude the listed firms in cities where hadn’t issued the lockdown policies. Table 6 shows the results.

**Table 6:** Effects of lockdown policy during COVID-19

Treatment group Control group	*CAR*			
	Energy firms 2020		Energy firms 2020	
	Energy firms 2019		Non-energy firms 2020	
	(1)	(2)	(3)	(4)
Treat*Post	0.054***	0.054***	0.025***	0.025***
	(0.011)	(0.011)	(0.008)	(0.008)
*Treat*	− 0.041***	− 0.041***	− 0.021***	− 0.022***
	(0.008)	(0.008)	(0.008)	(0.008)
*Post*	− 0.026***	− 0.026***	0.003	0.003
	(0.008)	(0.008)	(0.003)	(0.003)
*Size*		0.000		0.004***
		(0.003)		(0.001)
*Leverage*		0.016		− 0.004
		(0.018)		(0.009)
*Roe*		0.011		0.020
		(0.024)		(0.012)
*MB*		− 0.016**		− 0.001
		(0.007)		(0.002)
*Industry effects*	Yes	Yes	Yes	Yes
*Constant*	0.002	− 0.005	− 0.005	− 0.094***
	(0.007)	(0.069)	(0.016)	(0.035)
*Obs*	328	328	2700	2700
*R2*	0.096	0.104	0.024	0.027

The table shows the impact of lockdown policies during COVID-19 on market reaction. Dependent variable is cumulative abnormal returns (*CAR*) in a 5 days before and after lockdown date of cities in where listed firms is located. *Post* equals 1 for the period after lockdown date, and after the corresponding period of previous year in lunar calendar, and 0 otherwise. Adjusted robust standard errors are in parentheses. The definition of other variables is reported in Table 1. ***, **, and * is regarded as significance at the level of 1%, 5%, and 10%, respectively

We find that no matter comparing with energy firms in 2019, or non-energy firms in 2020, the coefficients of Treat*Post are significantly positive. It reveals that the implementation of the lockdown policy exerted the positive effect on *CAR* of energy firms. The lockdown policies in China could significantly increase the stock return of energy firms by 2.5%-5.4% on average.

## Conclusions

The uncertainty and decline of the global economy increasingly become the more serious during COVID-19. The international oil marker was hit by rude shocks, such as faltering demand of energy, rarely negative oil price, and the largest scale of cutting about oil, which attracted tremendous attention from firms, scholars, and policy makers. Moreover, given that China is a largest manufacturing country with the largest energy consumption, the volatility of energy market both home and abroad is critical for economic development. Hence, evaluations related to the market reaction of energy firms by COVID-19 are particularly important.

We investigate the market values of energy firms during COVID-19 in the context of China. First, based on the exogenous shocks of COVID-19, we show that COVID-19 significantly incurs an additional 2.7–10.6% decreased in CAR of energy firms. Second, based on the CSR performance from listed firms, we present strong evidence for resiliency of firms with better CSR performance prior to the pandemic when suffering market value losses during COVID-19. Lastly, lockdown policies during COVID-19 significantly increase the stock return of Chinese energy firms by 2.5–5.4% on average.

The results have three important implications. First, as the dynamic factor of economic development, energy firms suffered the more severe wealth losses than other industries because of the outbreak of COVID-19 pandemic. Given the importance of energy on China’ economy, the government should pay more attention on the risk resistance of energy industry, and try their best to ensure the supply and demand of energy, thereby stabilizing energy market. Second, our findings of the positive impact of CSR performance when the firm is hit by negative events, suggest that firms can pay more attention on CSR activities to build trust with stakeholders, eventually increasing risk resistance. Meanwhile, government should positively promote and encourage listed firms to fulfill social responsibility of and invest CSR activities a lot, thereby increasing corporate brand value. Third, by investigating the effectiveness of lockdown policies, we find that lockdown policies that restrict human mobility and economics activities have not resulted in great loss on economy, in turn reduced the losses of wealth by protecting the public health and maintaining social order. It demonstrates the positive effect of the control policy on economy, thereby contributing government to further supplement pre-warning system and related solution on public health emergency.

## Data Availability

The datasets generated during and/or analysed during the current study are available from the corresponding author on reasonable request.

## Abbreviations

COVID-19: Coronavirus disease 2019
CAR: Cumulative abnormal return
CSR: Corporate social responsibility
DID: Difference and differences
AR: Abnormal return
SIPO: State Intellectual Property Office
CSMAR: China Stock Market & Accounting Research
RKS: Rankins rating
CAPM: Capital assert pricing model

## Footnotes

1 Padhan and Prabheesh (2021) surveyed the existing research that focused on COVID-19 and concluded the three strands of economic consequence of COVID-19, including stock market, exchange rate, oil and energy market.

2 Wuhan in Hubei Province, China is the first city where the COVID-19 case were found in December 2019. To suppress the spread of COVID-19, the government of Wuhan issued the lockdown policy on January 23, 2020. It is this lockdown policy that almost all of the public in China was aware of COVID-19.

3 Rankins Agency of China issued the report on the review of listed firms’ corporate social responsibility reports from 2009 to 2018 on January 16, 2019. The related report can be found at: http://www.rksratings.cn/show-704-807-1.html.

4 The Spring Festival is the grandest festival in China, which is almost involved in all people in China. And the date of the Spring Festival in every year depends on the lunar calendar. Generally, the Chinese would be ready to welcome the coming of the Spring Festival, weeks in advance. At this moment, the public mainly arrange work based on the lunar calendar. Thus, for control groups that consists of energy firms in 2019, this paper assigns the variable ${Post}_{t}$ according to the same period in the 2019 lunar calendar.

5 Based on the assumption of event study method, the abnormal return equals zero without the occurrence of events.

6 We use raw value of CSR performance, or CSR performance divided by 100 to verify our findings, obtain the same results.

7 Fang et al. (2020) indicated the date and the type that all cities in China have issued the lockdown policies before February 20, 2020.

## References

[CR1] Albuquerque R, Koskinen Y, Zhang C (2019). Corporate social responsibility and firm risk: Theory and empirical evidence. Management Science.

[CR2] Albuquerque R, Koskinen Y, Yang S, Zhang C (2020). Resiliency of environmental and social stocks: An analysis of the exogenous COVID-19 market crash. The Review of Corporate Finance Studies.

[CR3] Alfaro, L., A. Chari, A. N. Greenland, and P. K. Schott. Aggregate and firm-level stock return during pandemics, in Real Time. NBER Working Paper Series No. 26950.

[CR4] Baker SR, Bloom N, Davis SJ, Kost KJ, Sammon MC, Viratyosin T (2020). The unprecedented stock market impact of COVID-19. The Review of Asset Pricing Studies.

[CR5] Bartik AW, Bertrand M, Cullen ZB, Glaeser EL, Luca M, Stanton CT (2020). The impact of COVID-19 on small business outcomes and expectations. Proceedings of the National Academy of Science of the United State of America.

[CR6] Chen H, Qian W, Wen Q (2021). The impact of the COVID-19 pandemic on consumption: Learning from high frequency transaction data. AEA Papers and Proceedings.

[CR7] Deng X, Kang J, Low B (2013). Corporate social responsibility and stakeholder value maximization: Evidence from mergers. Journal of Financial Economics.

[CR8] Ding W, Levine R, Lin C, Xie W (2021). Corporate immunity to the COVID-19 pandemic. Journal of Financial Economics.

[CR9] Fang H, Wang L, Yang Y (2020). Human mobility restrictions and the spread of the novel coronavirus (2019-nCoV) in China. Journal of Public Economics.

[CR10] He P, Sun Y, Zhang Y, Li T (2020). COVID-19’s impact on stock price across different sectors—An event study based on the Chinese stock market. Emerging Markets Finance and Trade.

[CR11] Huang S, Liu H (2021). Impact of COVID-19 on stock price crash risk: Evidence from Chinese energy firms. Energy Economics.

[CR12] Jia Z, Wen S, Lin B (2021). The effects and reacts of COVID-19 pandemic and international oil price on energy, economy, and environment in China. Applied Energy.

[CR13] Kong D (2012). Does corporate social responsibility matter in the food industry? Evidence from a nature experiment in China. Food Policy.

[CR14] Kong D, Shi L, Yang Z (2019). Product recalls, corporate social responsibility, and firm value: Evidence from the Chinese food industry. Food Policy.

[CR15] Lins KV, Servaes H, Tamayo A (2017). Social capital, trust, and firm performance: The value of corporate social responsibility during the financial crisis. The Journal of Finance.

[CR16] Liu S, Kong G, Kong D (2020). Causal effects of COVID-19 on air quality: Human mobility, spillover effect, and city connection. Environmental and Resource Economics.

[CR17] Maliszewska, M., Mattoo, A., Van Der Mensbrugghe, D., 2020. The potential impact of COVID-19 on GDP and trade: A preliminary assessment. In: World Bank Research Working Paper (9211).

[CR18] Padhan R, Prabheesh KP (2021). The economics of COVID-19 pandemic: A survey. Economic Analysis and Policy.

[CR19] Ren Y, Narayan S, Ma C (2021). Air quality, COVID-19, and the oil market: Evidence from China’s provinces. Economic Analysis and Policy.

[CR20] Si D, Li X, Xu X, Fang Y (2021). The risk spillover effect of the COVID-19 pandemic on energy sector: Evidence from China. Energy Economics.

[CR21] Wang, Y., D. Zhang, X. Wang, and Q. Fu. 2020. How Does COVID-19 Affect China’s Insurance Market? *Emerging Markets Finance and Trade*. 56(10): 2350–2362. 10.1080/1540496X.2020.1791074.

[CR22] Wei, R., X. Chen, and C. Chang. 2021. Does COVID-19 pandemic hurt stock prices of solar enterprise? *Economic Analysis and Policy*. 72: 41–57. 10.1016/j.eap.2021.07.011.

[CR23] Zhang W, Hamori S (2021). Crude oil market and stock markets during the COVID-19 pandemic: Evidence from the US, Japan, and Germany. International Review of Financial Analysis.

